# The edge orientation entropy of natural scenes is associated with infant visual preferences and adult aesthetic judgements

**DOI:** 10.1371/journal.pone.0316555

**Published:** 2025-02-26

**Authors:** Philip McAdams, Sara Svobodova, Taysa-Ja Newman, Kezia Terry, George Mather, Alice E. Skelton, Anna Franklin

**Affiliations:** 1 The Sussex Colour Group and Baby Lab, The School of Psychology, University of Sussex, Brighton, United Kingdom; 2 The School of Psychology, University of Sussex, Brighton, United Kingdom; 3 Nature and Development Lab, The School of Psychology, University of Sussex, Brighton, United Kingdom; Shahid Beheshti University, IRAN, ISLAMIC REPUBLIC OF

## Abstract

Statistical regularities of oriented edges in natural scenes, ‘edge co-occurrence statistics’, are associated with adults’ aesthetic responses, with greater preference for some images when the degree of randomness in the orientation of edges (Edge Orientation Entropy, EOE) across an image is relatively high. Here, we investigate whether this spatial image statistic is also associated with infants’ visual preferences. We measure infant looking time for images of building façades previously used to identify the relationship between EOE and adult aesthetic judgements. Twenty-six 4–9-month-old infants and 29 adults looked freely at pairs of the images. Infants and adults both looked longest at images where all edge orientations are about equally likely to occur (high 1st-order EOE), and at images with low correlation of edge orientations across the image (high 2nd-order EOE). Infant looking time and adult pleasantness judgements were also strongly related: infants looked longer at the building façades that adults liked. Our results suggest that even as young as 4-months, infants’ spatial vision is sensitive to edge co-occurrence statistics that are typical of natural scenes and faces, where edges are more evenly distributed across orientations. We discuss the implications for understanding the sensory component of adult aesthetic judgements, as well as the role of natural scene statistics in infant perception.

## Introduction

Decades of research has documented that even young infants look longer at some stimuli than others: for example, infants look longer at upright than inverted faces [1], vertical symmetry than horizontal [2], and red than green [3]. These ‘visual preferences’ have provided much insight into infants’ visual and perceptual systems, and the role of experience in perceptual development. The relationship between infants’ visual preferences and adults’ aesthetic judgements has also been investigated, and several studies have revealed that infants look longer at certain stimuli the more adults like them [4–7]. For example, infants look longer at faces that adults rate as attractive than unattractive [4], and look longer at preferred than disliked colours [5], biological motion [6], and art [7]. These associations between infant looking and adult liking do not necessarily indicate that infants have an ‘aesthetic’ response or ‘like’ certain stimuli, as infant looking can be driven by other factors such as interest, salience, or stimulation [5,8,9]. Rather, infants’ visual preferences are better understood as reflecting a sensory bias of the visual system, and the association with adult aesthetic judgements potentially provides further evidence that sensory biases contribute to aesthetic judgements later in life. One popular framework for understanding aesthetic judgements is as an interaction of sensory, emotive, and cognitive components [10]. Investigating infants’ visual preferences for aesthetic stimuli may help elucidate the sensory component, because the role of cognitive and emotional factors such as experience, conceptualisation, memory, knowledge, context, and culture is weaker in infancy relative to adulthood [7]. Establishing infants’ response to aesthetic stimuli is the first step in understanding how a mature aesthetic judgement develops. Beyond this, aesthetic stimuli provide a rich resource for further characterising infant perception.

Here, we further investigate the relationship between infants’ visual preferences and adult aesthetic judgements. We investigate whether infants look longer at building façades that adults find more pleasant, and whether spatial image statistics are similarly associated with infants’ and adults’ response. Buildings are a stimulus that most people encounter in their day to day lives, and often generate aesthetic responses [11]. Decades of research in Architectural Design and the emerging field of Neuroarchitecture [12], has revealed numerous features of buildings that affect people’s emotional, physical, and cognitive responses [13,14]. One line of research has focused on the low-level visual features and image statistics that could contribute to aesthetic judgements of architecture. For example, the fractal dimension [15,16] and Fourier spectral properties [17] of architecture have been found to affect peoples’ preference and well-being in spaces. In addition, one recent study has found that edge co-occurrence statistics are associated with adult aesthetic judgements for building façades [18].

Edge co-occurrence statistics measure the statistical regularities of edge orientations across images, where edges are defined as a steep change in luminance, e.g., at object boundaries [19]. Edge co-occurrence statistics play a role in perceptual processes such as contour grouping, and object occlusion, categorization and detection in complex scenes [20–23]. Grebenkina et al. [18] (see also [24]) measured edge co-occurrence statistics by calculating 1st-order and 2nd-order edge orientation entropy (EOE). First-order EOE measures the degree of randomness in the orientations of individual edges: an image where particular edge orientations are more frequent than others has low 1st-order EOE, whereas an image where no particular edge orientations are more frequent than others has high 1st-order EOE. Second-order EOE measures the degree of randomness in the relative orientations of pairs of edges: an image in which the orientations of edges are predictive of one another has lower 2nd-order EOE, and an image where the orientations of edges are independent has high 2nd-order EOE. Grebenkina et al. [18] found that the degree of randomness of oriented edges as measured by EOE predicted how pleasant and interesting adults rated the building façades: façades were more pleasant and interesting the greater the EOE. Low EOE façades have a range of orientated edges where certain orientations are more dominant than others (e.g., horizontal and vertical), representing a more simplistic façade, for example, as in Modern style office buildings. High EOE façades have a range of oriented edges where all possible orientations are more equally represented, and therefore will have more decoration and embellishments, for example, as in Renaissance architecture. EOE shares variance with curvature (high 1st-order and 2nd-order EOE images have more curves), which also relates to adult aesthetic judgement [18,25], although EOE is more strongly related than curvature to aesthetic judgements of building façades [25].

Here we use the same stimuli as Grebenkina et al. [18], and the infant preferential looking technique [7,26], to establish whether infants have a ‘visual preference’ for building façades with high EOE that corresponds to adults’ pleasantness judgements. The well-established infant preferential looking technique records infant looking times to pairs of stimuli, and as explained earlier, longer looking to one stimulus over the other is defined as a ‘visual preference’ indicating some form of sensory bias that does not necessarily imply that infants like the stimulus. Our question here is not whether infants like the same building façades as adults, but rather whether infants have a sensory bias for the building façades that adults find most pleasant, and whether EOE as a spatial image statistic can explain the variance in infants’ sensory bias.

Our study is motivated by two aims. First, the study aims to gain insight into the extent to which early sensory biases in infancy are associated with a mature aesthetic response in adulthood. Second, the study aims to gain further insight into the characteristics of infant perception. Whilst infant vision has been well characterised with simple stimuli that isolate chromatic and spatial properties [5,27], infants’ perception of complex stimuli such as art or natural scenes is less well characterised. Studies that investigate infants’ response to natural scenes are starting to provide insight into the role of top-down and bottom-up processing in infant perception [28], as well as further characterise infant eye-movements [29]. Other research has started to consider the extent to which infants are sensitive to the statistical regularities of natural scenes [30–32]. For example, one study suggests that infants are sensitive to natural scene texture statistics [30]; and infant color vision appears to be aligned with the distribution of chromaticities in natural scenes [32]. An investigation of infants’ response to van Gogh landscapes also found that infants’ visual preference for the landscapes could be partly accounted for by a combination of image statistics such as saturation and luminance contrast, as well as edge statistics such as edge density [7]. The current study aims to build on this research and investigate the role of EOE in infant perception.

In order to investigate whether infants look longer at buildings that adults find more pleasant, and to identify whether there is a contribution of EOE, the current study records infant and adult looking responses to pairs of building façade images from Grebenkina et al.’s [18] stimulus set. We also compute EOE for the images of building façades, which are controlled in the amount of luminance contrast. We eye-track infants and adults looking at pairs of the stimuli, and then correlate infant and adult looking time. We also correlate infant looking time with adult pleasantness ratings from Grebenkina et al. [18], and correlate 1st-order- and 2nd-order EOE with adult and infant measures. A series of multiple regressions investigates whether infant and adult responses are better accounted for by other spatial image statistics. The findings will establish whether the similarity between infant visual preferences and adult aesthetic judgements extends to architecture, and will also assess the role of EOE in infant perception.

## Method

### Participants

Twenty-nine infants aged between 18 and 39 weeks old (*M* =  28 weeks, *SD* =  6 weeks, 17 male) took part. Three infants’ data were excluded from analysis because either: (i) the number of trials completed was less than the inclusion rate (<20), due to fussiness; (ii) an accurate calibration was not completed; or (iii) the infant’s average looking time was classed as an outlier, calculated as 1.5 times less than or greater than the interquartile range. All infants were full term and weighed over 2500g at birth. Their parents reported no known neurological or visual conditions, or family history of color vision deficiency. Infants were opportunity sampled via social media and given a Baby Lab t-shirt for taking part in the study. There were 29 adult participants aged 18–56 years (*M* =  23 years, *SD* =  8.4 years, 6 male), with normal or corrected-to-normal vision, recruited via opportunity sampling from the University of Sussex student and staff body. Adult participants were compensated for their time at payment equivalent to the UK national minimum wage. Written informed consent was obtained from infants’ caregivers and adult participants; the study conforms to the tenets of the Declaration of Helsinki (other than pre-registration), and ethical approval was granted by the University of Sussex Sciences & Technology Cross-Schools Research Ethics Committee (ER/AES31/27) and from the European Research Council Executive Agency.

### Stimuli

**Fig 1:**
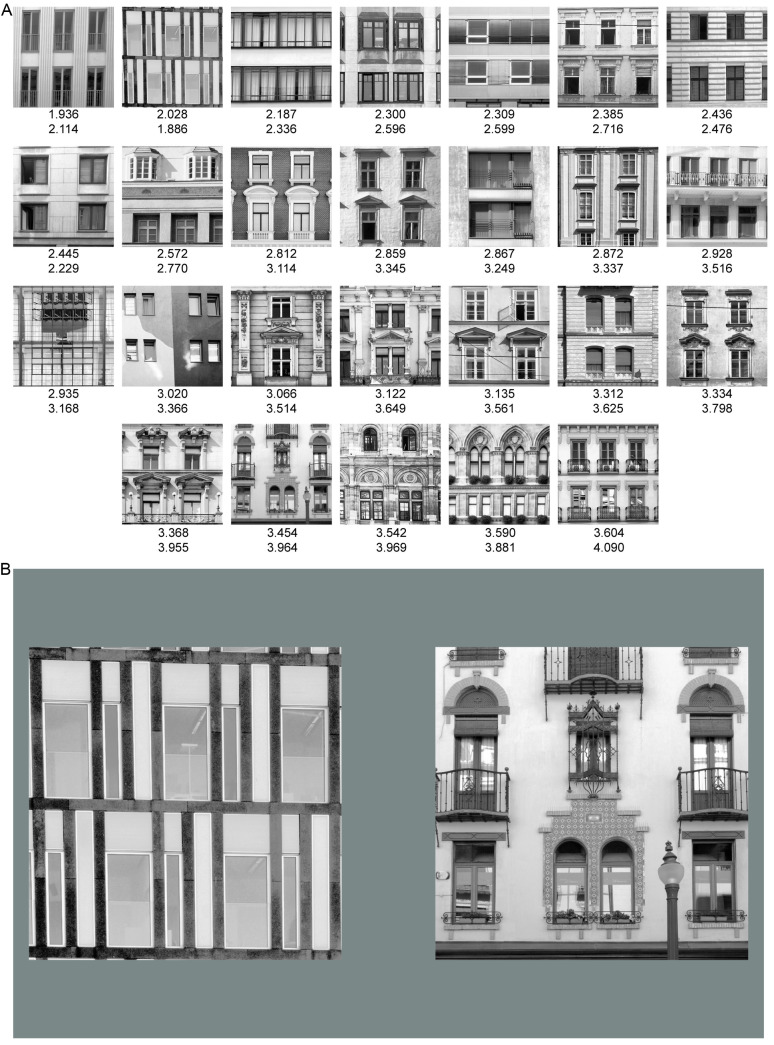
Stimuli and trial example. (A) Stimuli were sampled from a set of 26 cropped and square digital versions of building façades ranging from low to high 1st-order and 2nd-order edge orientation entropy. Façades are shown increasing in 1st-order edge orientation entropy from top-left to bottom-right, and with their corresponding edge orientation entropy values below each façade - 1st-order (top number) and 2nd-order (bottom number). (B) Stimuli were shown to participants paired on a grey background. Building façade images by Christoph Redies (https://osf.io/cxyj4/), licensed under CC BY 4.0 (https://creativecommons.org/licenses/by/4.0/).

Stimuli were 26 greyscale digital photographs of building façades, ranging from simple to highly ornamental, without faces, forms, figures, or writing, which might bias infant and adult looking (see Fig 1A). Stimuli were sampled from a stimulus set used in previous studies [18,33], and were photographed by Christoph Redies, mostly in Vienna and Berlin. Each image was cropped to select only two stories of a building. Images were prepared as described in Grebenkina et al. [18], and as described in brief below (see Image analysis). Stimuli were converted from color to greyscale using the ITU-R-601–2 luma transform [34], which weights the color channels according to their perceived luminosity, and the luminance histograms were equalised so that each image shared the same luminance distribution. Images were re-sized to 800 pixels x 800 pixels using bilinear scaling. Stimuli subtended a visual angle of 22.77° when shown as pairs with their inner edges 3.72° to the left and right of the centre point of the screen (see Fig 1B). Stimuli were displayed on a HP LP2480zx LCD Monitor (HP, Reading, UK) with a screen resolution of 1920 x 1200 pixels. Stimuli were shown on a neutral grey background (x =  0.29, y =  0.25, Y =  21.33 cd/m^2^). Eye-movements were recorded via an EyeLink1000 Plus system (SR Research, Ontario, Canada).

### Image analysis

Edge co-occurrence statistics were computed using the Python code for EOE as described by Redies et al. [24] (https://osf.io/bd8ma/). Briefly, images were filtered using a set of 24 oriented odd-phase Gabor filters representing one full rotation, akin to receptive fields in the human visual system [35,36], so the filters would respond maximally to edges in an image. Pixels with the highest 10,000 edge responses in the image were included in the EOE computation. The orientation of the maximum filter response at each pixel defined the edge orientation at that pixel. Orientation values were distributed into 24 bins, and 1st-order EOE was calculated from the resulting orientation histogram using Shannon entropy. Low 1st-order EOE indicates that particular orientations dominate in an image, and high values indicate that orientations are represented more equally. Second-order EOE was calculated by a pairwise comparison of all oriented edges. Orientation differences and pixel separations were binned, and 2nd-order EOE was calculated from the orientation-difference histogram at each distance bin using Shannon entropy. A summary entropy value was calculated from the average EOE for edge pairs separated by 20–240 pixels (see [24], for further details). Low 2nd-order EOE indicates that edge orientations at a given position can predict the orientations at other positions, whereas high values indicate that all orientation differences are equally likely to occur and edge orientations at one location are less predictive of other edge orientations at other locations (e.g., few parallel edges).

Additional image statistics (spectral slope, entropy, fractal dimension, horizontal symmetry, vertical symmetry, edge density, Pyramid Histogram of Oriented Gradients (PHOG) self-similarity, PHOG complexity, and lacunarity; see S1 Table, for definitions) were calculated on the greyscale image matrices. Image analyses were conducted using bespoke in-house algorithms [7,37,38], built-in MATLAB functions [39], and openly available algorithms [24,40,41]. Our code for image and data analysis, and the links to any open access code made available by others will be available from the corresponding author on reasonable request.

### Design and procedure

Each participant saw a random selection of 50 pairs of images. These were sampled from a total of 650 pairs which were created by each of the 26 stimuli being paired with every other stimulus twice with each stimulus in the pair appearing once on the left and once on the right. Infants and adults attended the experiment in-person, in a dimly-lit laboratory room, and were seated 50 cm from the display, at eye-level. Infants were seated in a car seat mounted on a chair. Infants watched a cartoon on the display during camera set up, and then completed a 4-point spatial calibration. Following this, each infant viewed 50 trials of randomly selected pairs of images (e.g., see Fig 1B), displayed for 5s each, with each infant viewing a different random selection of image pairs. Between each trial, an attention-getting stimulus was displayed, composed of a black and white, rotating, geometric pattern which looms and shrinks, lasting until the infant fixated it, to ensure the infants’ attention was centrally located at the start of a trial. For adult participants, the design was identical to that of the infants except that adults were asked to look freely at the images and to look centrally at the inter-trial attention getter to proceed to the next trial.

The eye-movement data was analysed using the DataViewer software (SR Research Ltd) to extract the time spent looking at the left or right area of interest which bounded each image. Looking time was measured as the total dwell time of all fixations in the area of interest, excluding the time spent making saccades. Fixations are determined as the periods between saccade offsets and onsets, detected using a velocity threshold of 40 degrees per second and an acceleration threshold of 8000 degrees per second squared. The looking time was then averaged across all presentations of each stimulus. The average number of fixations to each stimulus was also computed, although a preliminary analysis identified that average looking time and number of fixations across stimuli were highly correlated for infants (*r* =  0.96, *p* < .001) and for adults (*r* = .87, *p* < .001), therefore only looking time was selected for further analysis.

### Adult aesthetic ratings from Grebenkina et al. (2018)

Adults’ aesthetic judgements of the building façades from [18] were analyzed in the current study. Grebenkina et al.’s data was from 27 participants (*M* =  26.4, 14 male) who were asked to rate each building façade separately on three rating terms: interesting, pleasantness, and harmonious. We select the pleasantness measure for the current study because it is representative of the fundamental hedonic value of aesthetic evaluations [42], and found it correlated highly with the interest dimension in Grebenkina et al.

## Results

### Relationship of infant and adult looking times

**Fig 2:**
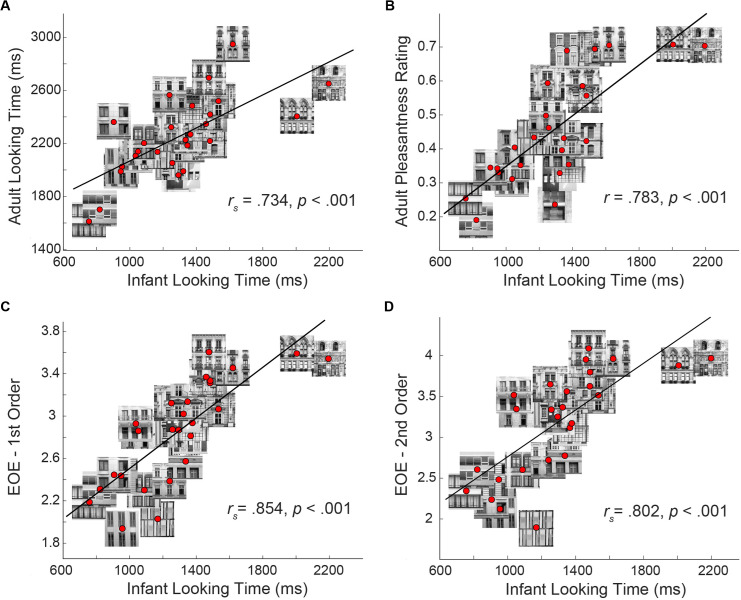
Scatterplots of the relationships with infant looking time. The relationships with infant looking time (averaged across participants) and other variables, across the 26 building façades. Thumbnail images have been centred on the red data points to give insight into the visual characteristics underlying the relationships. (A) The relationship between infant and adult looking time (averaged across participants). (B) The relationship between infant looking time and adult pleasantness ratings (averaged across participants). (C) The relationship between infant looking time and 1st-order edge orientation entropy. (D) The relationship between infant looking time and 2nd-order edge orientation entropy.

For both infants and adults, the average looking time at each building façade was calculated (dwell time, excluding saccades – see Method), averaging across all presentations of that façade on left and right sides. This average looking time measure was then averaged across infants or adults for each façade. Infant and adult looking time across the façades were strongly related: infants looked longer at the building façades which adults looked longer at, *r*_*s*_ =  0.734, *n* =  26, *p* < .001, BF_10_ =  492 (see Fig 2A).

### Relationship of infant looking time and adult pleasantness ratings

Adults’ pleasantness ratings from Grebenkina et al. [18] were converted to a scale ranging from 0 to 1. Infant looking time and adult pleasantness scores across the façades had a strong relationship, where infants looked longer at façades which adults rated as more pleasant, *r* =  0.783, *n* =  26, *p* < .001, BF_10_ =  9438 (see Fig 2B).

### Relationship of EOE with looking time and pleasantness ratings

Infant looking time and 1st-order and 2nd-order EOE were strongly related: infants looked longer at façades with higher EOE, 1st-order: *r*_*s*_ =  0.854, *n* =  26, *p* < .001, BF_10_ =  12991; and 2nd-order: *r*_*s*_ =  0.802, *n* =  26, *p* < .001, BF_10_ =  1966 (see Fig 2C and 2D). Adult looking time and 1st-order and 2nd-order EOE revealed a strong relationship where adults looked longer at façades with higher EOE, 1st-order: *r* =  0.610, *n* =  26, *p* < .001, BF_10_ =  44; and 2nd-order: *r* =  0.558, *n* =  26, *p* < .001, BF_10_ =  16. Adult pleasantness and 1st-order and 2nd-order EOE also revealed a strong relationship, 1st-order: *r* =  0.651, *n* =  26, *p* < .001, BF_10_ =  114; and 2nd-order: *r* =  0.617, *n* =  26, *p* < .001, BF_10_ =  51.

### Comparison of EOE correlations with infant and adult measures

To establish whether infant looking times correlated more strongly with EOE than adult looking times, we computed confidence intervals for the correlation coefficients and their differences using bootstrap methods (as described in [43]). Participants were sampled independently with replacement from each group (infants and adults, preserving the dependency among dyads of observations) to create bootstrap samples. We then computed the two correlation coefficients based on the bootstrap samples, calculated and recorded the difference between the correlations, and repeated this 10,000 times. We then used the distribution of bootstrap differences to derive a confidence interval. The difference between the strength of the correlations with EOE for infants’ and adults’ looking time is significant for 1st-order EOE (difference: 0.29 [CI =  0.046, 0.562], *p* = .009), and 2nd-order EOE (difference: 0.28 [CI 0.006, 0.578], *p* = .044). First-order EOE also correlated significantly more strongly with infant looking time than adult pleasantness rating (difference: 0.17 [CI 0.028, 0.373], *p* = .016); however, there was no effect for 2nd-order EOE(difference: 0.14 [CI −0.032, 0.342], *p* = .108).

### Commonality analysis

Commonality analysis (CA) was conducted to examine the shared variance among adult pleasantness ratings, EOE, and infant looking time. CA decomposes the total variance explained by predictors into unique and shared components by conducting a full regression model and comparing it with partial models for each predictor [44]. The unique contribution of each predictor is calculated by subtracting the unique variance explained by the other predictor from the total variance explained by the full model, while the common contribution, or shared variance, is calculated by subtracting the unique contributions of each predictor from the total variance explained by the full model. First and 2nd-order EOE were analysed separately.

An overall multiple regression model indicated that 73.4% of the variance in infant looking time can be explained by the combined influence of 1st-order EOE and adult pleasantness ratings (*F*(2,23) = 35.49, *p* < .001, *R*^*2*^_adj_ = .734, BF =  1351656). Adult pleasantness ratings uniquely explained 12.2% of the variance, 1st-order EOE uniquely explained 14.2% of the variance, and the shared variance among all three variables was 49.2%.

Similarly, 69.2% of the variance in infant looking time was explained by the combined influence of 2nd-order EOE and adult pleasantness ratings (*F*(2,23) =  29.079, *p* < .001, *R*^*2*^_adj_ = .692, BF =  29029). Adult pleasantness ratings uniquely explained 17.5% of the variance, 2nd-order EOE uniquely explained 10.3% of the variance, and the shared variance among all three variables was 43.9%.

### Contribution of other image statistics to infant looking time

To investigate whether the relationship between infant looking and EOE could be explained by other image statistics that correlate with EOE, we also conducted multiple regression analyses with a range of additional image statistics as predictors: spectral slope, entropy, fractal dimension, horizontal symmetry, vertical symmetry, edge density, PHOG self-similarity, PHOG complexity, and lacunarity (see S1 Table for definitions). We conducted the regressions on 1st-order and 2nd-order EOE separately as these two variables are highly correlated (*r* = .972, *p* < .001). Variables were first screened for normality, linearity, and homoscedasticity, then converted into z-scores; then for variable selection, we used a backward elimination model, where simultaneously entered predictors are removed sequentially if the *p*-value of the regression coefficient is >  0.1 [45]. To assess the individual importance of predictors, we also calculated variance inflation factors (VIFs) to measure how much of the variance of an estimated regression coefficient is increased because of collinearity [46]. We removed predictors with high VIFs, which shared variance with the strongest predictor, until all VIFs were <  1.25 (i.e., <  20% variance explained by other predictors, and indicative of no multicollinearity issues [46,47] (see [7] for additional description).

A backward multiple linear regression with 1st-order EOE and the additional image statistics significantly predicted infant looking time, *F*(2, 23) =  31.451, *p* <  0.001, adj. *R*^2^ = .71, BF_10_ =  52588. Two variables added to the model: 1st-order EOE (*β* =  0.666, *p* < .001), and edge density (*β* =  0.340, *p* = .008), with 1st-order EOE being the most significant predictor. We repeated this analysis for 2nd-order EOE and the additional statistics finding that the model significantly predicted infant looking time, *F*(3, 22) =  18.630, *p* < .001, adj. *R*^2^ = .68, BF_10_ =  6585. Three variables added to the model: 2nd-order EOE (*β* =  0.572, *p* < .001), edge density (*β* =  0.333, *p* = .014), and horizontal symmetry (*β* =  0.224, *p* = .074), with 2nd-order EOE being the most significant predictor.

Equivalent backward linear regression analyses on adult pleasantness ratings were conducted with 1st-order and 2nd-order EOE. The model with 1st-order EOE significantly predicted adult pleasantness scores, *F*(2, 23) =  16.133, *p* <  0.001, adj. *R*^2^ = .55, BF_10_ =  562. Two predictors added significantly to the model: 1^st^-order EOE as the strongest predictor (*β* =  0.487, *p* = .003) and edge density (*β* =  0.432, *p* = .007). The 2nd-order EOE model also significantly predicted adult pleasantness score, *F*(2, 23) =  14.99, *p* <  0.001, adj. *R*^2^ = .53, BF_10_ =  369, with two equivalently strong predictors: 2nd-order EOE (*β* =  0.458, *p* = .005) and edge density (*β* =  0.459, *p* = .005).

## Discussion

The current study aimed to investigate whether infants and adults look longer at building façades that adults find more pleasant, and to identify whether there was a contribution of edge co-occurrence statistics to infants’ and adults’ responses. We found that infants and adults looked longer at the building façades which adults rated as more pleasant. We also found that EOE explained a large amount of the variance in how long infants and adults looked at, and how pleasant adults found, certain façades. Commonality Analysis identified that there was a large percentage of shared variance among infant looking time, adult pleasantness ratings, and EOE (almost half of the variance was shared between the three variables). Multiple regression analyses that included several other spatial image statistics that draw on image features, such as the spatial frequency, contrast, and symmetry of the images, established that EOE predicted the most variance in infant and adult responses compared to these other image properties investigated.

The finding that infants look longer at the building façades the more pleasant adults find them extends prior research which has also found associations of infant looking and adult aesthetic judgements for other types of stimuli [4,5,7]. One interpretation of these associations is that the visual system has sensory biases that partially govern both infant looking and adult aesthetic judgement. In support of the argument that infants and adults are similar in their response to EOE, we find a large amount of shared variance between infant looking, adult pleasantness ratings, and EOE. This could potentially provide support for the theory that early sensory biases provide the basis for aesthetic responses to form later in development. In the current study, both infant and adult looking and adult pleasantness judgments are biased to high EOE. This bias for high EOE stimuli could also be related to complexity since EOE relates to the amount of variety in the stimulus, which is a type of complexity, and stimuli generally appear more complex the higher the EOE. Although the nature of the relationship between complexity and aesthetics appears to vary according to the type of complexity investigated (e.g., number or variety of elements, organization, or symmetry [48]), the relationship between EOE and adult pleasantness ratings is consistent with prior research which finds that beauty increases with the variety of elements (see [48]). The findings of the current study potentially provide support for a Neuroaesthetic theory which proposes that images which mirror the statistical structure of natural scenes (such as images with high EOE [24]) are more preferred aesthetically because the human visual system is adapted to process these statistics efficiently through phylogeny and/or ontogeny [49–52]. The neural representation of edge co-occurrence statistics in natural scenes is thought to be a sparse one which reduces redundancy, allowing better edge detection, maximising neural resources and minimising metabolic costs ([35,52–56], cf. [57]). This efficient coding has been theorised to contribute to adult aesthetic preference, and it has been proposed that stimuli which reflect the statistical regularities of natural scenes are more efficiently processed, which in turn, increases their aesthetic value [58,59]. For example, people tend to prefer some stimuli with a spectral slope and fractal dimension characteristic of natural scenes [60,61]. One possibility is that preference for high EOE is due to certain real-world stimuli such as landscapes, clouds, and trees typically having high 1st-order and 2nd-order EOE, and faces having high 1st-order and moderately high 2nd-order EOE [24]. Adaptation to particular natural scenes with high EOE, such as those containing faces, could reduce sensitivity to high EOE, making images with high EOE more comfortable to view and therefore more liked [49]. Further research is needed to test this ‘adaptation’ hypothesis. Viewed through this framework, the relationships between EOE and adults’ pleasantness judgements do not suggest that aesthetic value is in the stimulus, but rather it arises from how efficiently that stimulus is processed by our visual systems as a result of adaptation to natural scenes.

The current study also extends the limited research on infant perception of natural scene statistics. First and 2nd-order EOE explained more of the variance in infants’ looking than adults’ looking. This might be expected given infants’ greater reliance on bottom-up processing of scenes [62]. It seems plausible that infants would pay relatively more attention to low-level visual properties of scenes than adults due to less experience, conceptualisation, and memory of certain spaces. The façades are likely to be perceived more abstractly by infants, resulting in a more sensory response, whereas adults are likely to have a greater influence of cognitive factors such as memories and associations triggered by the façades. That infants have a bias to certain edge co-occurrence statistics reveals the capabilities of the infant visual system. The Goldilocks model of infant attention proposes that infants allocate attention to stimuli with a level of complexity that is “just right” for their ability [63]. Although infants have relatively immature visual systems in many ways, our finding that infants look longest at the images with the highest EOE, identifies that infants’ visual systems can manage this level of complexity. This could be considered when designing for infants so that design (e.g., book illustration) is optimized and “just right” for infants’ visual abilities [64].

The neural basis of EOE is thought to be in orientation-selective cells in the visual cortex and their long-range horizontal and feedback connections [65–67], and such neural circuits are thought to be present by at least 4-months [68–70]. Therefore, our finding that infant visual preferences are associated with EOE is consistent with this neural basis. A sensory bias for stimuli with high EOE in infancy could be functional as it would draw infants’ attention to elements of natural scenes with relatively high 1st or 2nd-order EOE that are evolutionarily important, such as faces [24]. EOE is also useful for adult categorization of scenes [23] and contributes to other perceptual processes in adults, such as object occlusion [22]. Therefore, now that the current study has established that infants’ response is associated with EOE, further research can investigate whether a sensitivity to EOE is functional for infants’ perception and categorization of objects and scenes [23,71,72].

Another question for further research is the extent to which infants’ sensory bias for high EOE building façades is associated with their ‘visual diet’. We consider it unlikely that the infants’ sensory bias for high EOE building façades is due to how novel or familiar certain architectural styles are to them. For example, the infant participants lived in a city with a mix of architectural styles; it is unclear the extent to which young infants attend to building façades in their daily life; and we have no evidence that the infant participants associated the stimuli with buildings rather than viewing them as abstract patterns. Another possibility is that the sensory bias for high EOE building façades is due to greater novelty or familiarity of high EOE stimuli in general, or greater attention to certain stimuli with high EOE (such as faces) [24]. The infant participants will have been exposed to a range of natural and carpentered scenes which vary in their EOE, before taking part in the experiment, and although we do know that faces are dominant in infants’ ‘visual diet’ [73], it is currently unknown whether high EOE would be more familiar or novel than low EOE stimuli to the infants. However, further research which quantifies the EOE of infants’ ‘visual diet’ with head-mounted cameras [74], which does this for infants living in different environments, and which then measures those infants’ sensory biases for images with a range of EOE, would enable direct investigation of the extent to which infants’ sensory biases are associated with environmental experience. The current investigation, in identifying infants’ sensory bias for high EOE building façades, paves the way for further research to address these theoretically interesting questions.

## Conclusion

In conclusion, we reveal a striking similarity between infants’ visual preferences and adults’ aesthetic judgements: infants look longer at building façades that adults judge to be pleasant. We also identify that edge co-occurrence statistics are associated with how long infants and adults look at building façades, not only how pleasant adults find them. The findings contribute to our understanding of the role of sensory processes in aesthetic judgements. The findings also suggest that, even as young as 4-months of age, infants’ perception is responsive to the edge co-occurrence statistics that are typical of natural scenes and human faces.

## Supporting information

S1 Table Additional image statistics definitions and code used. (DOCX)
