# Supplementary material for: The edge orientation entropy of natural scenes is associated with infant visual preferences and adult aesthetic judgements
Source: PLoS One. 2025 Feb 26;20(2):e0316555. doi: 10.1371/journal.pone.0316555 (PMC11864518; doi:10.1371/journal.pone.0316555)
Supplement: S1 Table — (DOCX) [file pone.0316555.s001.docx]

**S1 Table**

**S1 Table. Additional image statistics definitions and code used.**

| **Image Statistic** | **Definition** |
| --- | --- |
| Spectral Slope | Slope of the function relating Log Fourier amplitude (averaged over orientation) to Log spatial frequency (Field, 1993). A steeper slope indicates scenes characterised by higher contrast at low frequencies (large, prominent features) and less contrast at high frequencies (fine details). Code: Mather, (2020). |
| Fractal dimension | Geometric properties of a scene, measures the degree to which self-similar patterns at different scales fill the image space. High values indicate greater complexity (space-filling), lower values indicate less complexity. Code: Box-counting (Moisy, 2022). |
| Edge Density | The concentration of edges in a scene. Higher edge density indicates more detailed textures, objects, and scene content. Low edge density indicates sparsity of edges. Code: (Redies et al., 2017). |
| Entropy | Describes the randomness of scene content by measuring the degree to which pixel intensities at any one location predict nearby values. High values indicate unpredictable scene content and more complexity. Low values indicate more redundancy/uniformity. Code: Built-in Matlab Shannon ‘entropy’ function (The MathWorks Inc, 2024) |
| Symmetry | Vertical symmetry flips the image left to right, and horizontal symmetry flips the image up to down, and then calculates the mean square difference of the flipped versions to the originals, on a pixel-by-pixel comparison. Code: Built-in Matlab functions ‘immse’, ‘flipud’, and ‘fliplr’ (The MathWorks Inc, 2024). |
| PHOG Self-similarity | How similar the gradient patterns are within different parts of an image. It compares the histograms of oriented gradients (HOGS) in the whole image with HOGS in sub-regions 1/64th the size of the whole image to see how consistently they appear across the image. Code: Redies et al. (2012). |
| PHOG Complexity | The overall complexity of an image based how much variation there is in the pixel intensities across scales of an image. A higher mean gradient indicates a more complex image, while a lower mean suggests a simpler one. Code: Redies et al. (2012). |
| Lacunarity | The spatial distribution of gap sizes across an image indicating the degree of non-uniformity of an image's texture (Mandelbrot, 1982; Plotnick et al., 1996). Code: gliding box-counting technique (Reuter, 2016). |
